# c-Kit^+^ Cells in Adult Salivary Glands do not Function as Tissue Stem Cells

**DOI:** 10.1038/s41598-018-32557-1

**Published:** 2018-09-21

**Authors:** Mingyu Kwak, Ninche Ninche, Sabine Klein, Dieter Saur, Soosan Ghazizadeh

**Affiliations:** 1 Department of Oral Biology & Pathology, Stony Brook University, Stony Brook, NY 11794 USA; 2 Department of Internal Medicine, Technical University of Munich, München, Germany

## Abstract

A rare population of salivary gland cells isolated based on c-Kit immunoreactivity are thought to represent tissue stem cells since they exhibit the most robust proliferative and differentiation capacity *ex vivo*. Despite their high promise for cell-based therapies aimed at restoring salivary function, the precise location and *in vivo* function of c-Kit^+^ stem cells remain unclear. Here, by combining immunostaining with c-KitCre^ERT2^-based genetic labeling and lineage tracing in the adult mouse salivary glands, we show that c-Kit is expressed in a relatively large and heterogeneous cell population that consists mostly of differentiated cells. Moreover, c-Kit does not mark ductal stem cells that are known to express cytokeratin K14. Tracking the fate of *in vivo*-labeled c-Kit^+^ or that of K14^+^ cells in spheroid cultures reveals a limited proliferative potential for c-Kit^+^ cells and identifies K14^+^ cells as the major source of salispheres in these cultures. Long-term *in vivo* lineage tracing studies indicate that although c-Kit marks at least two discrete ductal cell lineages, c-Kit^+^ cells do not contribute to the normal maintenance of any other cell lineages. Our results indicate that c-Kit is not a reliable marker for salivary gland stem cells, which has important implications for salivary gland regenerative therapies.

## Introduction

**Figure 1:**
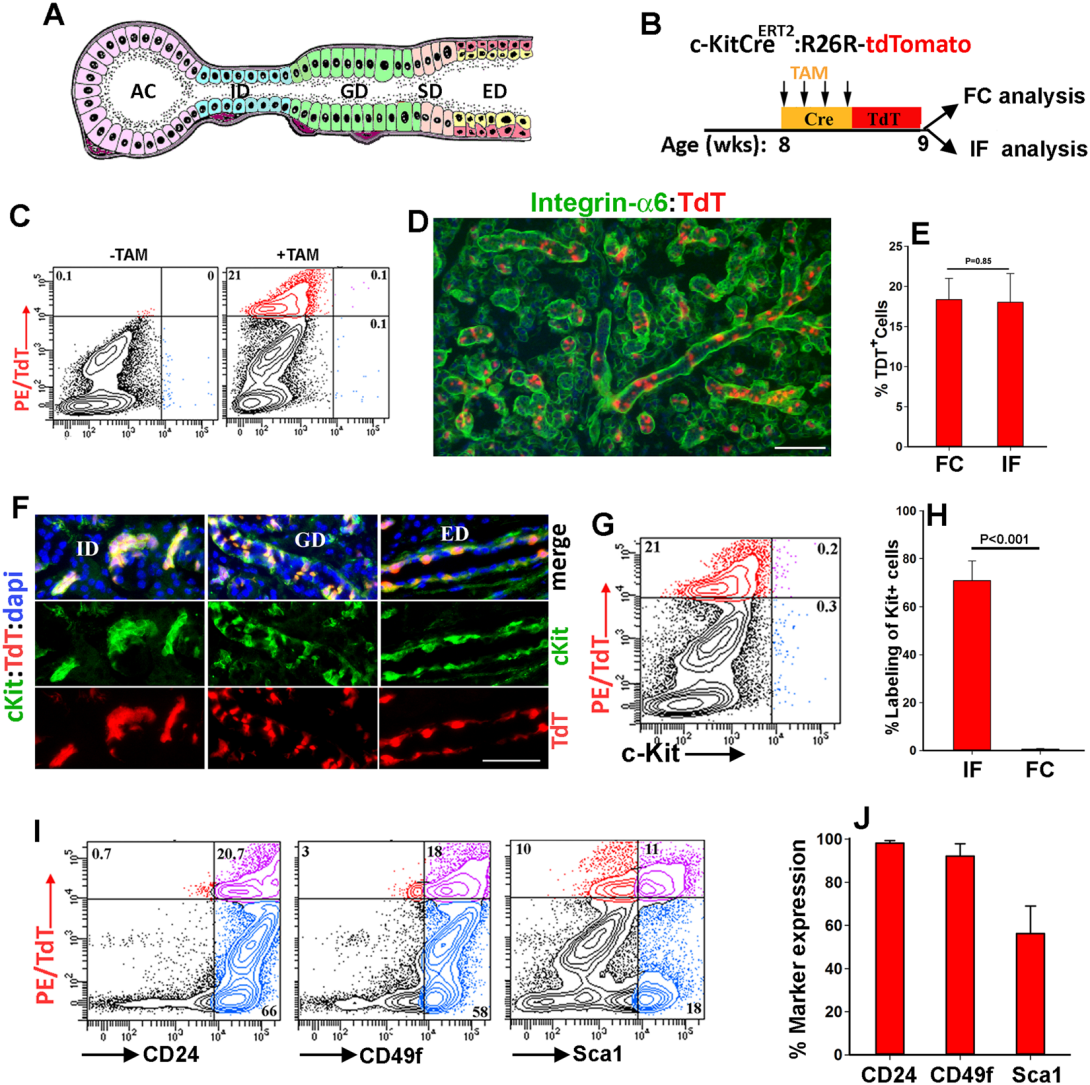
Genetic labeling reveals broad expression of c-Kit in salivary glands. (**A**) Schematic of the submandibular gland (SMG) structure in rodents. AC, acini; ID, intercalated duct; GD, granular duct; SD, striated duct; ED, excretory duct. (**B**) Strategy used for genetic labeling of c-Kit-expressing cells with tdTomato (TdT) in adult mice (8 wks of age, n = 5 including 3 female and 2 male mice). Tamoxifen (TAM) was administered for 4 consecutive days and glands were harvested 3 days later and analyzed by flow cytometry (FC) and immunofluorescence microscopy (IF). (**C**) TdT expression in total population of SMG cells in control (−TAM) and labeled mice (+TAM). (**D**) IF images of TdT-labeled SMGs immunostained for Integrin α6 (green). Scale bar = 100 μm. (**E**) Quantification of TdT labeled cells in total population of SMG cells using FC or IF. (**F**–**H**) c-Kit immunoreactivity of TdT-labeled cells in tissue sections (**F**) or single cell suspensions of SMGs (**G**). Single channels and merged images of various ductal compartments are shown in F. Nuclear blue staining is dapi. Scale bar = 50 μm. Graph in (**H**) shows the percentage of TdT-labeled cells immunoreactive to c-Kit antibody in tissue sections (IF) and in single cell suspensions (FC) (n = 3). (**I**,**J**) Expression of surface markers including CD24, CD49f or Sca1 by TdT^+^ cells. For all graphs, values are the mean ± SD with n = 5 unless indicated otherwise.

Major salivary glands (SGs) are complex lobular structures composed of at least seven differentiated parenchymal cell lineages organized into three differentiating epithelial tissues including acini, ducts, and myoepithelial cells^1^. Saliva is secreted from acini (secretory end pieces) and flows sequentially into intercalated (ID), granular (GD; specific to rodent submandibular glands)^2^, striated (SD), and excretory (ED) ducts that further modify and deliver saliva to the oral cavity (Fig. 1A)^3^. Although SGs are capable of repair and regeneration, various conditions including radiation therapy for head and neck cancers, auto-immune diseases, and aging can cause irreversible damage to SGs, severely affecting oral and overall health^4^. Currently, cell-based regenerative therapies aimed at the functional restoration of SGs are being developed^5–7^, and a subset of SG cells isolated based on their c-Kit immunoreactivity has been most effective in restoring salivary hypofunction in a mouse model of radiation-induced injury^8–11^.

c-Kit is a receptor tyrosine kinase that was initially described as a surface antigen detected on hematopoietic stem and progenitor cells^12^. Subsequently, c-Kit was found to mark progenitor cells in non-hematopoietic tissues including SGs^13–15^. Following the initial report by Hisatomi *et al*. describing flow cytometric isolation of a c-Kit^+^/Sca1^+^ cell population with high proliferative potential from adult mouse submandibular glands (SMG), several laboratories have used c-Kit with various combinations of cell surface markers including Sca1, CD29, CD49f and CD24 to enrich for stem cells in either human or mouse SMGs^8,9,16,17^. The reported frequency of c-Kit^+^ cells in these cell preparations is extremely low, ranging between 0.06% to 1%^8,9,13,17^. However, transplantation of as few as 300 mouse c-Kit^+^ cells or 1200 human c-Kit^+^ cells isolated from spheroid cultures of SMGs is sufficient to significantly improve salivary secretory function in a mouse model of radiation-induced injury^8,10,11,16^. Subsequent studies demonstrated that a subset of SMG cells isolated based on combinations of 4 surface markers (Lin^−^CD24^+^c-Kit^+^Sca1^+^) had the highest spheroid-forming efficiency in culture and displayed a robust multilineage regenerative capacity when transplanted into irradiated mouse SMGs^9^. Despite this additional enrichment, the isolated cell population remained heterogeneous and whether an individual c-Kit^+^ cell within this population undergoes self-renewal and multilineage differentiation could not be determined^9^.

During the embryonic development of SMGs, c-Kit is broadly expressed by the end bud epithelial cells and is required for survival and proliferation of progenitors during branching morphogenesis and proacinar differentiation^18^. At birth, however, c-Kit expression is shifted from proacinar cells to ductal cells, where it continues to be expressed to adulthood^19^. In histological sections of adult mouse SMGs, c-Kit immunopositive cells are detected in various ductal compartments including the IDs, SDs, and EDs at much higher frequencies than those reported by flow cytometry analyses^8,9,20,21^. Although the broad tissue distribution of c-Kit^+^ cells has hindered efforts to map the precise location of c-Kit^+^ stem cells, several studies have suggested that these stem cells reside in the EDs^5,9,21^. Transcriptional profiling of the Lin^−^CD24^+^c-Kit^+^Sca1^+^ cell fraction has revealed high levels of basal cell cytokeratins K14 and K5 in this population, implying that c-Kit^+^ stem cells express K14/K5^9^. Furthermore, co-immunostaining of tissue sections for c-Kit and K5 has shown co-localization of these two markers in a small subset of ductal cells in the ID and the ED where c-Kit^+^ and K14/K5^+^ cells are juxtaposed^21,22^. K14 has been identified as a marker of the lineage-restricted ductal stem cells that reside in the basal layer of the ED and at the ID/GD junction^20,22^. Long-term lineage tracing of K14^+^ stem cells, however, did not reveal any lineal relationship between c-Kit^+^ and K14^+^ cells in the ID. Although similar studies have not been performed in the ED, it is clear that K14^+^ cells in the ED do not function as multi-lineage stem cells in the adult gland^20,22^. Recent long-term lineage tracing of several cell populations including ductal and acinar cells have indicated that homeostasis of the adult salivary gland does not rely on multipotent stem cells^22–24^. Despite the high promise of c-Kit as a marker for SG stem cells and its potential application for cell-based therapies, the *in vivo* location and function of c-Kit^+^ stem cells remain unclear.

Here, we first verified the frequency and distribution pattern of c-Kit^+^ cells in all major SGs of adult mice through genetic labeling and immunostaining. We then used an inducible genetic lineage-tracing approach to investigate the fate of *in vivo*-labeled c-Kit^+^ cells in spheroid cultures and during homeostatic renewal of the adult SMG. Our analysis did not identify c-Kit as a marker for salivary gland stem cells.

## Results

### c-Kit is broadly expressed in the major salivary glands of adult mice

To clarify the discrepancy between c-Kit immunoreactivity of tissue and single cell suspensions of adult SMGs, we used a genetic approach to label c-Kit-expressing cells with a fluorescent protein and then compared the frequency and distribution pattern of the genetically-labeled cells with that of c-Kit-immunoreactive cells in the SMG. A well-characterized *c-KitCre*^*ERT2*/+^ mouse line with a tamoxifen (TAM)-inducible form of Cre recombinase driven by an endogenous *c-Kit* locus was crossed with R26R-tdTomato (TdT) reporter strain carrying a floxed stop codon between the ubiquitously expressed Rosa26 promoter and a gene encoding TdT, a variant of red fluorescent protein^25,26^. TAM was administered to bi-transgenic mice (8 wks of age, 3 females and 2 males) for four consecutive days to efficiently label c-Kit-expressing cells^25^ and three days later, SGs were removed for immunofluorescent and flow cytometry analysis (Fig. 1B). Analysis of TdT-labeled cells in tissue sections or single cell suspensions of SMGs showed that TAM administration induced TdT expression in about 20% of total population of SMG cells (Fig. 1C–E). The TDT-expressing cells were predominantly mapped to the salivary ducts (Fig. S1). To verify the cell specificity of c-Kit-driven Cre recombination of the R26R-TdT locus, histological sections and single cell suspensions of TdT-labeled SMGs were immunostained for c-Kit protein (Fig. 1F,G). Immunofluorescent microscopy revealed a strong concordance between TdT expression and c-Kit protein in tissue sections of SMGs, with a distribution pattern consistent with previous reports (Fig. 1F)^20,21^. TdT^+^ c-Kit^+^ cells were detected throughout the salivary ducts and were either organized into uniformly labeled cell clusters in the ID or distributed more sporadically in the larger salivary ducts (Fig. 1F). In contrast, flow cytometry analyses of single cell suspensions prepared from the contralateral gland using the same anti-c-Kit antibody (clone 2B8) showed no correlation between TdT labeling and c-Kit immunopositivity (Fig. 1G,H). The minimal immunoreactivity of c-Kit-expressing cells in single cell suspensions of SMGs was verified using a different anti-c-Kit antibody (clone Ack2), and it was consistent with previous flow cytometric analysis indicating a frequency for c-Kit immunopositive cells ranging from 0.058%^8^ to 0.97%^13^ in total population of SMG cells. Flow cytometry analysis of TdT-labeled SMG cells for expression of other parenchymal cell surface markers that are often used in combination with c-Kit to enrich for stem cells, including CD24, CD49f (pan-epithelial, Figs 1D and S2), and Sca1 (a marker expressed in salivary ducts, Fig. S2), showed that almost all TdT-expressing cells in SMGs are epithelial cells, and more than 50% express Sca1 (Fig. 1I,J). These data indicated that c-Kit is expressed in a relatively large and heterogeneous epithelial cell population in SMGs. Moreover, the minimal concordance between TdT expression and c-Kit-immunopositivity indicated that c-Kit epitopes are altered in single cell suspensions of SMGs.

**Figure 2:**
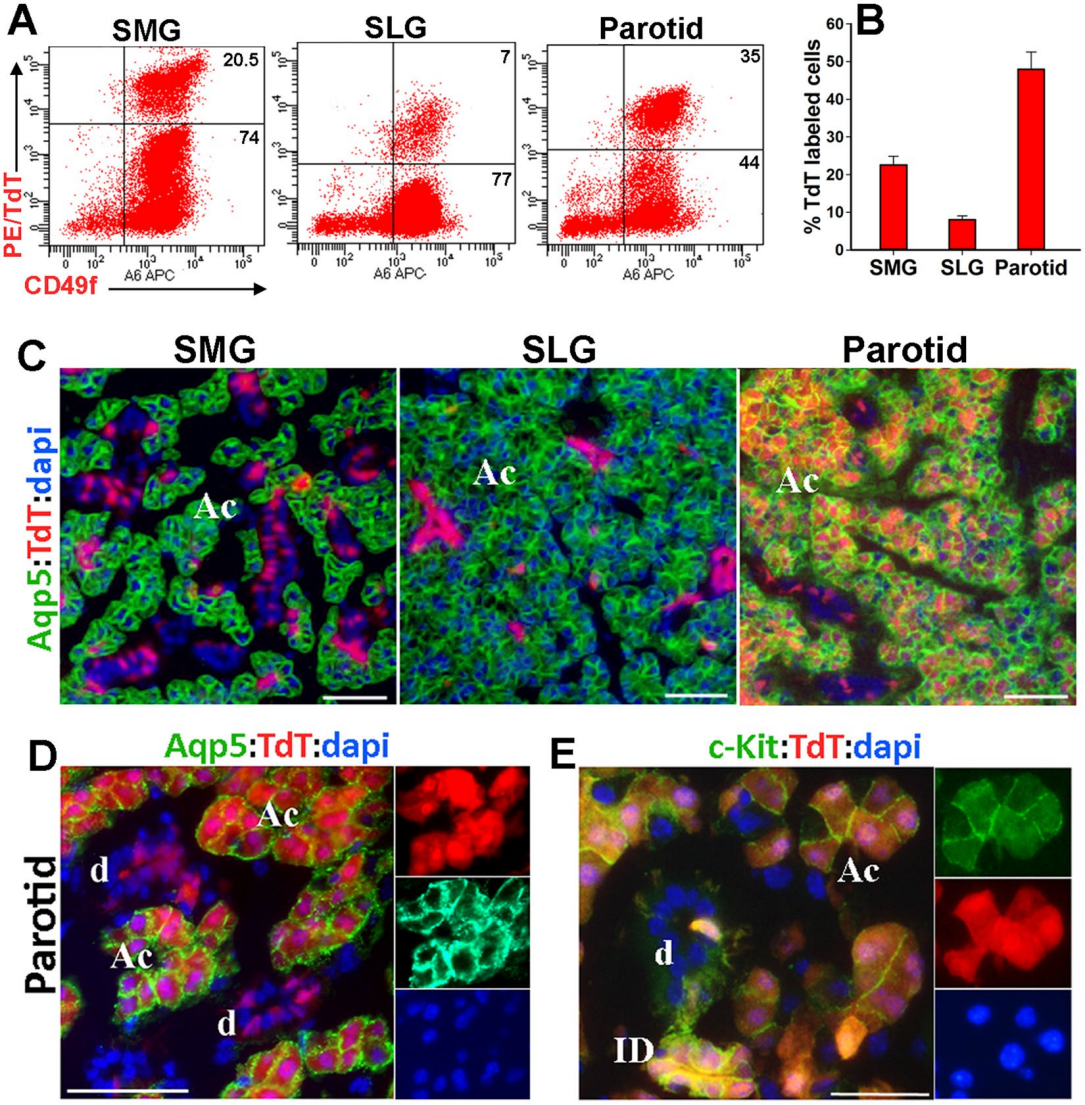
Differential c-Kit expression pattern in major salivary glands. (**A**,**B**) Percentages of TdT-labeled CD49f^+^ cells in submandibular (SMG), sublingual (SLG) and parotid glands isolated from mice described in Fig. 1B. Values in graph are mean ± SD from 3 female mice. P < 0.001 for all pair-wise comparisons. (**C**) Representative images of TdT-labeled SMG, SLG and parotid glands immunostained for Aqp5 (n = 3). (**D**,**E**) IF images of parotid acini immunostained for either Aqp5 (**D**) or c-Kit. (**E**) Both single channels and merged images are shown. AC, acini; ID, intercalated duct; and SD, striated duct. Nuclear blue staining is dapi. Scale bars = 50 μm (**C**,**D**) and = 25 μm (**E**).

Analysis of TdT-labeled cells in other major SGs of bi-transgenic mice revealed a striking difference in the frequency and distribution pattern of c-Kit-expressing parenchymal cells in parotid glands when compared to SMG and sublingual glands (Figs 2A,B and S3). The acinar to ductal ratio is comparable for the parotid and sublingual glands (9 acinar to 1 ductal) and much higher than that in the SMG (6 acinar to 4 ductal)^27^. Despite this, the number of TdT-labeled cells in parotid glands was significantly higher than the other major salivary glands (Fig. 2A,B). Immunostaining for aquaporin 5 (Aqp5), a water channel expressed exclusively in acinar cells^28^, revealed widespread TdT expression in parotid acini (Fig. 2C,D). Immunostaining for c-Kit protein verified concurrent expression of TdT and c-Kit in acinar cells and appropriate localization of c-Kit on the surface of acinar and ID cells (Fig. 2E). Overall, the widespread expression of c-Kit in the differentiated compartments of SGs such as GDs, SDs, and parotid acini indicate that c-Kit is not a specific marker for SG stem cells.

### c-Kit and K14 mark distinct cell populations

**Figure 3:**
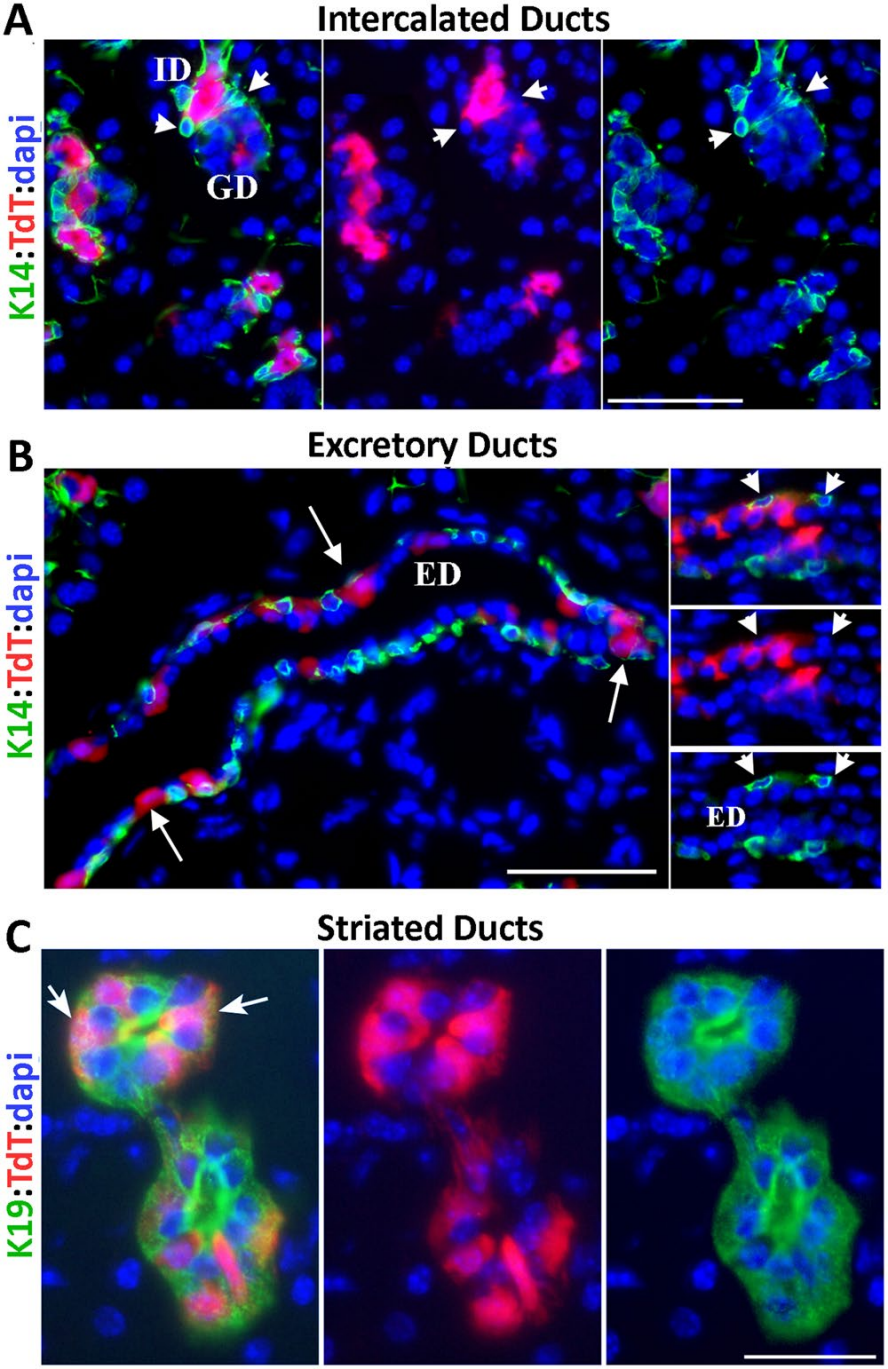
c-Kit does not mark K14^+^ stem cells. (**A**-**B**) Representative images of TdT-labeled SMG of c-KitCre^ERT2^:R26R-TdT mice stained for K14 (green) showing no overlap between TdT and K14 in the intercalated (**A**) and excretory ducts (ED) (n = 5). (**B**) Arrowheads point to the location of K14^+^ ductal stem cells. Arrows point to flask-like morphology of TdT^+^ cells in the ED. Scale bars = 50 μm. (**C**) Images of striated ducts stained for ductal marker K19 (green) showing flask-like morphology of TdT-labeled cells (noted by arrows) in this differentiated compartment. Nuclear blue staining is dapi. Scale bars = 25 μm. Both merged and single channels are included.

Previous studies have suggested that c-Kit^+^ stem cells co-express basal cytokeratins (K14/K5) and that they reside in EDs^9,21^. To more precisely map the position of c-Kit-expressing cells with respect to K14^+^ ductal cells, sections of TdT-labeled SMGs were immunostained for K14. In SGs, K14 marks ductal stem cells in EDs and, IDs, as well as myoepithelial cells^22,29^. Fluorescent microscopy analysis of the EDs and IDs showed no overlap between TdT and K14 expression (n = 480 K14^+^ ID cells and n = 340 K14^+^ basal ED cells) (Fig. 3A,B arrowheads and Fig. S4). In the IDs, TdT was expressed in luminal cells juxtaposed to K14^+^ ductal stem cells (Fig. 3A), confirming our previous studies indicating that c-Kit and K14 mark two discrete cell lineages^22^. In the EDs, c-Kit-expressing cells (TdT^+^) were scattered among the K14^+^ basal cells, however, they displayed a distinct columnar, flask-like morphology (Figs 3B and S4, arrows). The morphology and distribution pattern of c-Kit^+^ cells in the EDs were comparable to that of c-Kit^+^ cells in highly differentiated GDs and SDs (Figs 3C and S1). In these compartments identified by K19 expression, c-Kit-expressing cells were scattered among the principal ductal cells and displayed a unique flask-like morphology with narrow and long basolateral projections similar to what has been described for “Tuft” cells (Figs 3C and S4, arrows)^30^. Tuft cells are highly specialized chemosensory cells found in various epithelial tissues that play a key role in regulating innate immunity^31^. Overall, these findings indicate that K14 and c-Kit mark distinct cell populations, and they underscore the heterogeneity of cKit-expressing cells in salivary ducts.

### Contribution of c-Kit^+^ and K14^+^ cells to salisphere formation in cultures of SMGs

**Figure 4:**
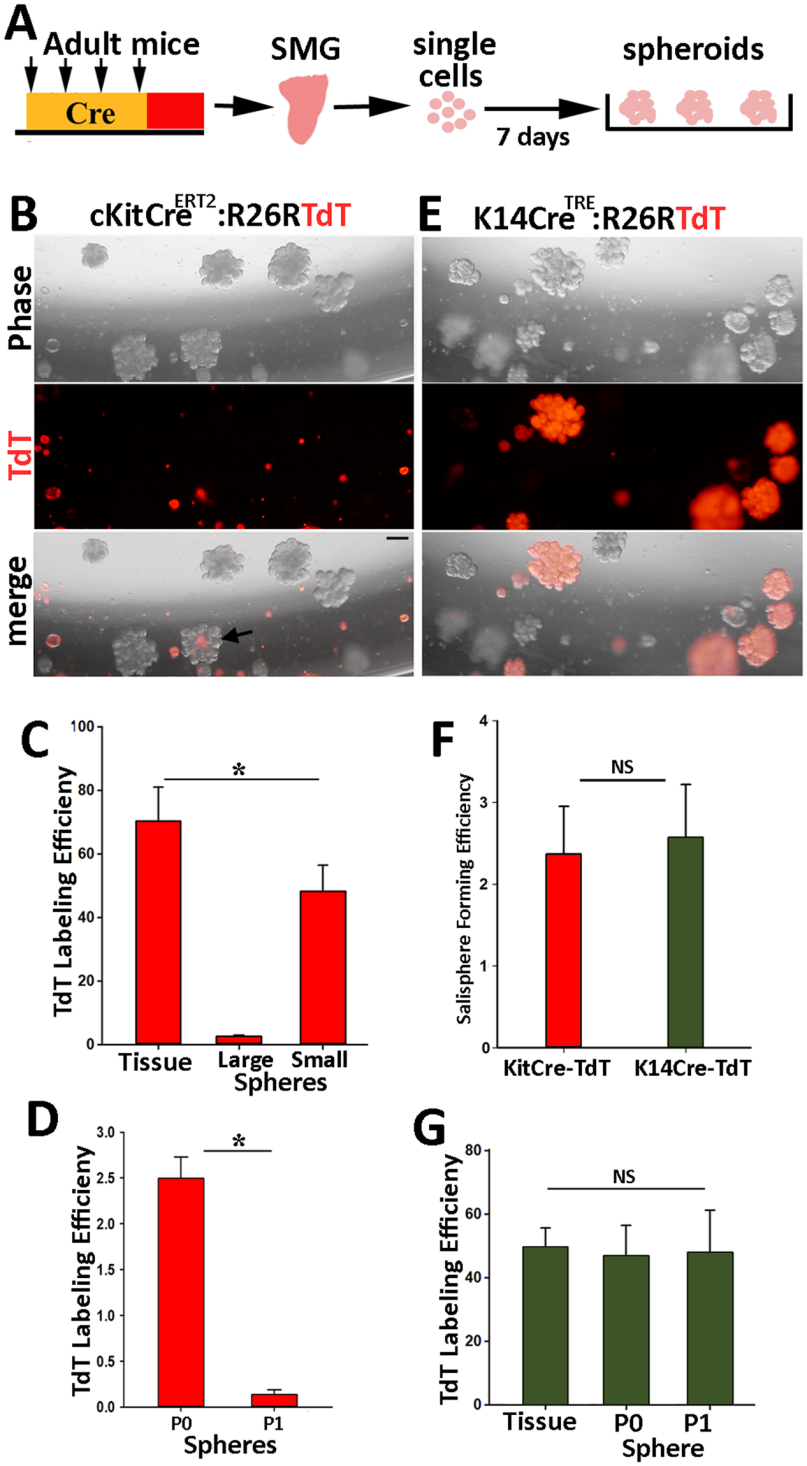
c-Kit^+^ cells do not contribute to formation of salispheres in culture. (**A**) Strategy used for tracking the fate of *in vivo*-labeled cells in spheroid cultures of total population of SMG cells prepared from mice at 8–10 wks of age. (**B**) Representative phase and fluorescent images of spheres established from SMG cells of cKitCre^ERT2^-TdT mice after 8 days in culture (n = 4 glands). Arrow points to a salisphere containing a small number of TdT^+^ cells. Scale bars = 200 μm. (**C**) TdT-labeling efficiency of c-Kit^+^ cells in tissue (SMG) and in primary spheroid cultures. Spheres were scored based on their size with large being >0.2 mm and small being <0.2 mm in diameter. (**D**) The percentage of large TdT-labeled spheres in primary (P0) and secondary (P1) cultures established from c-KitCre^ERT2^:R26R-TdT mice. (**E**) Images of salispheres established from SMG cells of K14Cre^TRE^:R26RTdT after 8 days in culture. Scale bars = 200 μm. (**F**) Salisphere forming efficiency of total population of SMG cells isolated from transgenic mouse models. (**G**) TdT-labeling efficiency of K14^+^ stem cells *in vivo* (tissue) and that of spheres in primary and secondary spheroid cultures. In all graphs, values are expressed as mean ± SEM from at least 3 female mice. ^*^P < 0.01, NS = not significant.

The expression of c-Kit by differentiated cells in salivary ducts and parotid acini does not exclude the existence of a small subset of c-Kit^+^ cells that may function as SG stem cells. Since the robust proliferative and differentiation capacity of a subset of SG cells in spheroid cultures has been attributed to c-Kit^+^ cells^9,17,32^, we traced the fate of *in vivo*-labeled TdT-expressing cells in spheroid cultures established from total population of SMG cells. TAM was administered to *cKitCre*^*ERT2*/+^: R26RTdT mice (8–10 wks of age) to label c-Kit^+^ cell *in vivo*. Three days later, both SMGs were removed, one gland was processed for immunofluorescent analysis to quantify the efficiency of TdT-labeling in c-Kit-expressing cells, the other gland was dissociated into a single cell suspension to generate salispheres (Fig. 4A). Analysis of cultures from TAM-treated and non-treated littermates showed a comparable sphere-forming efficiency of about 2.5% ± 0.8% for spheres measuring ≥0.2 mm in diameter at 7 days post-seeding (Fig. S5A). Interestingly, despite a fairly high efficiency of *in vivo* labeling for c-Kit^+^ cells at 72% ± 12% (Fig. 4B,C), over 95% of salispheres formed in culture did not express TdT (n = 1320 from 3 mice; Fig. 4B,C). In fact, although TdT-labeled cells accounted for about 20% of cells initially present in culture, the majority failed to grow to spheres larger than 0.1 mm in diameter (Fig. 4B,C). Moreover, upon passage, the proportion of TdT^+^ spheres further declined to 0.3% ± 0.4% (n = 1560; Figs 4D and S5B). Failure of TdT-labeled cell in contributing to salispheres formed in cultures of SMGs suggested that a different cell population contributes to salispheres. Given that K14 marks an actively cycling stem cell population in adult SMGs^22^, we wondered if this population was the source of salispheres. A similar labeling strategy was applied to *K14Cre*^*TRE*^: R26RTdT mice to mark K14^+^ cells with TdT *in vivo* and assess their sphere-forming capacity *in vitro* (Fig. 1E–G). Analysis of spheroid cultures showed that the overall salisphere-forming efficiencies of SMG cells were comparable between the two mouse models (Fig. 4F). However, in spheroid cultures established from K14Cre^*TRE*^:R26RTdT mice, approximately half of the large and highly branched salispheres were uniformly labeled with TdT, suggesting they were descendants of a single K14^+^TdT^+^ cell (Fig. 4E). More importantly, the labeling efficiency of K14^+^ ductal stem cells within the tissue corresponded directly to the percentage of TdT^+^ spheres in culture, indicating that K14^+^ stem cells were the major source of salisphere formation in culture (Fig. 4G). In addition, when these primary spheroid cultures were dissociated and re-plated, the proportion of TdT-labeled spheres remained unchanged, confirming the self-renewal ability of K14^+^ cells in culture (Fig. 4G). Overall, these data demonstrate that SG stem cells contribute to the formation of salispheres in culture, however, no subset of c-Kit^+^ cell population is capable of extended growth in salisphere cultures.

### c-Kit^+^ cells do not contribute to the maintenance of other cell lineages during homeostasis

**Figure 5:**
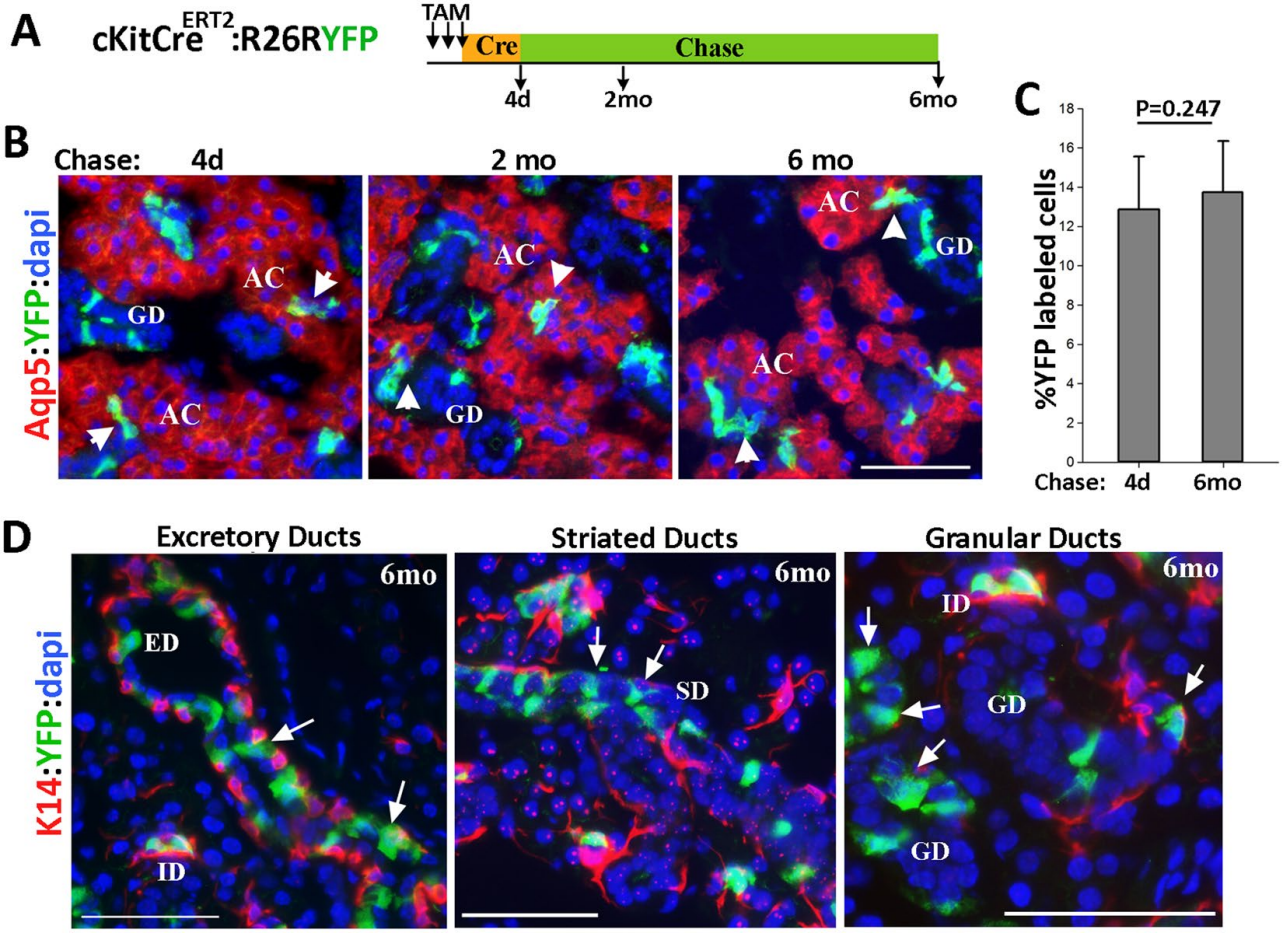
Lineage tracing of c-Kit^+^ cells in the adult SMG. (**A**) Experimental strategy for lineage tracing of c-Kit^+^ cells in adult mice. (**B**) Representative fluorescent images of SMGs at 4 days, 2 months, and 6 months after the last dose of tamoxifen (TAM). Sections were co-stained for YFP (green) and Aqp5 (red) to identify acini. Blue nuclear staining is dapi. Arrowheads indicate the YFP-labeled intercalated ducts. (**C**) The percentage of YFP-labeled cells after 4 days and 6 months of chase. Values are expressed as mean ± SEM using 10 images (400X)/group. Differences are insignificant *P* = 0.247. (**D**) Images of salivary ducts at 6 months of chase co-stained for YFP (green) and K14 (red) showing persistence of sporadically distributed YFP-labeled cells in the larger ducts. AC, acini; ID, intercalated ducts; GD, granular ducts; ED, excretory ducts; SD, striated ducts. Images shown are from female transgenic mice and representative of both sexes (n = 5 mice). Scale bar = 50 μm.

A recent lineage tracing study has demonstrated that c-Kit^+^ ID cells do not contribute to acini^24^, consistent with previous studies indicating that acini are maintained independent of ductal progenitors^23^. However, this study offered limited analysis of c-Kit^+^ cells in other ductal compartments^24^. Therefore we used a similar lineage tracing approach to more quantitatively analyze the fate of all subsets of c-Kit^+^ cells in adult SMGs. TAM was administered to 6-wk old *c-KitCre*^*ERT2*/+^: R26RYFP mice for three consecutive days to pulse label c-Kit^+^ cells with YFP, and then the number and position of RosaYFP-labeled cells were analyzed and compared over 6 months (Fig. 5A). Analysis of YFP-labeled cells at 4 days or following a 2- or 6-month chase period showed a similar pattern of distribution for YFP-labeled cells (Figs 5B and S6). YFP-labeled cells remained restricted to the ID cells and the tuft-like cells in larger ducts during the entire chase period. Moreover, quantitative analysis of YFP-labeled cells showed no significant change in their number during the 6-month chase period (Figs 5B,C and S6). As expected, the descendants of Rosa-YFP cells in the ID cells did not expand into acini over the 6-month chase period (Fig. 5B arrowheads, n = 260 ID/acini junctions), confirming previous studies^23,24^. More importantly, however, even after a 6-month chase period, the YFP-labeled cells with flask-like/columnar morphology were detected in the EDs, SDs and GDs (Fig. 5D, arrows). Co-immunostaining of SMGs for YFP and K14 verified the persistence of YFP-labeled cells juxtaposed to the K14^+^ progenitors in the ED, suggesting no lineal relationships between c-Kit^+^ and K14^+^ cells (Fig. 5D). The stable frequency of YFP^+^ cells in salivary ducts indicated that c-Kit^+^ cells were not replaced at the population level by unlabeled progenitor or stem cells. Overall, our lineage tracing experiments revealed the presence of at least two distinct cell lineages marked by c-Kit, one in the ID and the other in the larger ducts that are maintained independent of K14^+^ ductal stem cells. Moreover, a lack of contribution of c-Kit^+^ cells to any other cell type during homeostasis is not consistent with the behavior of stem cells^33^.

## Discussion

Tissue stem cells are defined as self-renewing cells that generate at least one type of differentiated descendant during tissue homeostasis^33–35^. The identification of c-Kit as a marker for SG stem cells was based on *ex vivo* characterization of a c-Kit-immunoreactive cell population isolated from the adult mouse SMGs^8,9,13,17^. Our genetic labeling and lineage tracing studies were aimed at providing direct evidence for the existence of c-Kit^+^ stem cells in the adult gland. However, the broad expression of c-Kit in SGs, the lack of contribution of c-Kit^+^ cells to any other differentiated cell lineages during tissue homeostasis, and their limited proliferative potential in spheroid cultures were all inconsistent with the existence of c-Kit^+^ stem cells in adult SGs.

The minimal contribution of genetically-labeled cKit-expressing cells to salisphere formation revealed by our *in vitro* fate analysis did not validate the widely-accepted notion that c-Kit^+^ stem cells are the major producers of salispheres in cultures of adult SMGs^9,10,17,32^. Previous studies have relied on c-Kit immunoreactivity to enrich for a rare cell population in single cell suspensions of adult SMGs that displayed a robust salisphere-forming capacity in culture^8,9,11,13^. However, the reported frequencies of c-Kit immunopositive cells in single cell suspensions were too low (ranging from 0.05% to 1%) and inconsistent with the broad c-Kit expression in ductal cells observed in histological sections of SMGs^8,9,13,20,21^. To overcome the potential problems stemming from unreliable c-Kit immunoreactivity, we used a transgenic mouse line in which endogenous activity of the *c-Kit* locus dictated expression of Cre and recombination of a Rosa locus resulted in the expression of a fluorescent protein, thus enabling purification, mapping, and lineage tracing of c-Kit cells without relying on c-Kit immunoreactivity^25,26^. The cell-type specificity of Cre-mediated recombination of *R26R-TdT* locus was confirmed by immunostaining for endogenous c-Kit protein in tissue sections, however, TdT expression was not in concordance with c-Kit immunoreactivity in single cell suspensions of SMGs. These data strongly suggest that the enzymatic tissue digestion of salivary glands can damage or alter externally exposed c-Kit epitopes as shown in other tissues^36^. Therefore, the results of studies using c-Kit immunoreactivity to isolate SG stem cells should be interpreted with caution as the isolated cell populations do not represent c-Kit^+^ cells. It is worth noting that even when a combination of four surface markers (Lin^−^CD24^+^cKit^+^Sca1^+^) was used to enrich for SG stem cells, the isolated stem cell population remained heterogeneous with a salisphere-forming efficiency of less than 3%^9^. Furthermore, transcriptome profiling of the isolated cell population indicated high levels of K5 and K14 transcripts, suggesting inclusion of K14^+^ cells in the sorted population^9^. In the absence of validation of c-Kit expression at single cell levels in the sorted cell population, it is not clear whether salisphere formation by this heterogeneous population was solely attributable to the c-Kit^+^ cells or to contaminating K14^+^ ductal stem cells or other progenitor populations. Indeed, another study has shown that SMG cells enriched for high levels of pan epithelial cell surface markers CD24 and CD29 were highly efficient in salisphere formation, but further purification based on c-Kit immunoreactivity did not improve the proliferative capacity of this population^32^.

Our data clearly demonstrate that c-Kit and K14 mark two distinct cell populations in the adult SMGs. Previous studies have suggested that a population of K14^+^c-Kit^+^ multipotent stem cells resides in the basal layer of EDs^5,9,21^. However, the lack of co-localization of K14 protein (antibody) and TdT expression (c-Kit expressing cells), the distinct morphology of c-Kit^+^ cells when compared to K14^+^ basal cells in the ED, the differential contribution of K14^+^ cells and c-Kit^+^ cells to salispheres in culture (50% for K14^+^ cells vs. 2.5% for c-Kit^+^ cells in primary spheres and 50% vs 0.3% in secondary cultures), and the long-term lineage tracing analysis of c-Kit cells in this study and that of K14^+^ cells in our previous studies showing a lack of lineal relationship between these cell populations^20,22^ are all inconsistent with the existence of a c-Kit^+^K14^+^ cell population in the adult gland. Although a K14^+^Kit^+^ embryonic progenitor population has been identified during early development of SMGs, the lineage of K14^+^ and Kit^+^ cells may diverge early during development. This is supported by a recent study indicating that c-Kit and K14 are inversely regulated in response to retinoic acid during development^37^. In the present study, we relied on the Cre-lox system in transgenic mice, the “gold standard” for addressing the problems of *in vivo* cellular origins^38^. The genetic labeling did not appear to have an adverse effect on the proliferative potential of c-Kit^+^ cells in culture since neither tamoxifen administration nor expression of Cre and TdT altered the overall salisphere-forming efficiency of SMG cells. Furthermore, a similar approach using K14Cre^TRE^:TdT mice in which the efficiency of Cre recombination in ductal stem cells was about 50% (Fig. 4G) demonstrated uniform TdT expression in 50% of salispheres formed from total population of SMG cells indicating that K14^+^ ductal stem cells are the major contribute to salisphere formation in cultures. One caveat of our genetic labeling is that not every c-Kit^+^ cell was labeled with TdT and therefore we cannot directly rule out the possibility of a bias against Cre activation in the presumptive multipotent stem cell population in EDs. However, the persistence of genetically labeled c-Kit^+^ cells in EDs revealed by our long-term *in vivo* lineage tracing argues against this possibility. Given the relatively rapid turnover rate in EDs^20^, if there was a c-Kit^+^K14^+^ stem cell population in which *c-KitCre* locus was not active, then the YFP-labeled c-Kit^+^ cells should have been gradually replaced by descendants of this unlabeled stem cell population, resulting in a gradual decline in the number of YFP-labeled cells during the 6-month chase period. Clearly, the persistence of YFP-labeled cells in EDs indicates that the lineage of c-Kit^+^ cells is related neither to the K14^+^ stem cells, nor to the presumptive K14^+^Kit^+^ stem cells.

Our long-term lineage tracing of c-Kit^+^ cells confirmed a recent lineage tracing analysis of c-Kit^+^ ID cells demonstrating that c-Kit^+^ ID cells do not contribute to acini in submandibular and sublingual glands^24^. Classical nuclear tracking studies have suggested that IDs harbor stem cells for both differentiated ductal and acinar cells^1,18,39^. We have recently identified the ductal stem cells in IDs that contribute to the maintenance of the K19^+^ differentiated cell lineage in GDs^22^. However, since c-Kit is uniformly expressed by all ID cells localized between the ductal stem cells and acini, the lack of expansion of lineage-traced cells into acini does not support these earlier studies, but rather is consistent with recent studies indicating that acini homeostasis relies entirely on the proliferative capacity of existing acinar cells^23^. Although neither populations of c-Kit^+^ cells contribute to any other differentiated cell lineages in the adult SMGs, the persistence of genetically-labeled cells in both IDs and larger ducts reveals two distinct ductal cell lineages. The maintenance of these two lineages is independent of K14^+^ ductal stem cells and underscore the complexity of ductal cell lineages in adult SGs. In the larger ducts, c-Kit is expressed in luminal cells with a distinct barrel-shaped or flask-like morphology and distribution pattern similar to that described for highly specialized tuft cells^30^. Although c-Kit expression in tuft cells must be further investigated, c-Kit signaling may be involved in the regulation of various secretory, chemosensory, and immune regulatory functions attributed to tuft cells^30,31^. In addition to salivary ducts, c-Kit expression is most prominent in the acini of parotid glands. The basis for differential expression of c-Kit in acini between parotid and SMGs is not clear because in rodents, both glands are composed of mostly serous acini^3^. However, recent studies have indicated functional differences in ion transporter activity between the parotid and SMG acinar cells^27^. Clearly, the functional significance of c-Kit signaling in these highly specialized cell populations requires further investigation.

In summary, the results presented here indicate that c-Kit is not a reliable marker for SG stem cells and argues against the existence of a Kit^+^ stem cell population in SGs. Furthermore, given the therapeutic potential of salisphere-derived cells in reversing irradiation-induced hyposalivation^9,10,17^, the identification of K14^+^ stem cells as an expandable source of SG cells in culture has important implications for cell-based therapies aimed at restoring SG function.

## Methods

### Animals

Transgenic c-Kit-Cre^ERT2+/−^ mice in which a tamoxifen-inducible form of Cre recombinase is driven from an endogenous c-Kit locus have been described previously^25,26^. These mice were kept on C57Bl/6 background. Cre reporter strains R26R-tdTomato (stock# 007914) and R26R-YFP (Stock# 006148) were purchased from Jackson Laboratory (Bar Harbor, Maine). Mice were genotyped by polymerase chain reaction using mouse genomic DNA from tail biopsy specimens. Selective labeling in c-Kit^+^ cells in adult mice was induced by intraperitoneal injection of tamoxifen (TAM, 1 mg/50 ul of peanut oil/mouse; Sigma Aldrich, St. Louis, MO) for 3 or 4 consecutive days as described by previous studies^26^. Transgenic K14-Cre^TRE/+^ mice with Cre expression regulated by doxycycline was used to induced selective labeling of K14^+^ cells as described previously^22^.

Adult male and female mice aged between 8–10 weeks were used in all experiments with the exception of long-term lineage tracing studies in which Tam was administered at 6 weeks of age. The care and experimental use of animals were approved by the Institutional Animal Care and Use Committee (IACUC protocol#287271) of the Stony Brook University, Stony Brook, New York, USA. Animals were maintained in the Stony Brook University-Health Science Center Division of Laboratory Animal Resources, an AAALAC-accredited center, and housed in groups of up to five mice per cage under maximum isolation with fresh food and water and regular cleaning. All animal experiments were performed in accordance with institutional guidelines set forth by the State University of New York.

### SG Cell Preparation and Spheroid Cultures

SMGs were harvested, the attached fat and connective tissues were carefully removed, and the glands were separated with one processed for histology and the other for cell preparation. For cell preparation, SMGs were dissociated by mechanical and enzymatic digestion (0.025% Collagenase, 0.05% hyaluronidase, 1U/ml dispase) to single cell suspensions as described previously^20^. Ten thousand SMG cells from this single cell suspension were re-suspended in 40 μl media, mixed with 60 μl Matrigel (BD Biosciences, San Jose, CA) on ice, seeded evenly around the periphery of a well of a 12-well plate (in triplicates) to ease counting of spheres. Plates were placed at 37 °C for 20 min to allow Matrigel to solidify. Cell-Matrigel mixture was covered with 1 ml of salisphere growing media containing epidermal growth factor (20 ng/ml), fibroblast growth factor-2 (20 ng/ml), N2 supplement, insulin (10 μg/ml), dexamethasone (1 μM), 5% fetal bovine serum and 10 μM ROCK inhibitor Y-27632 (Sigma) and half of the media was changed every 2–3 days^40^. Cultures were grown for 7–8 days before being analyzed by fluorescent phase-contrast microscopy. To establish secondary cultures, primary salispheres were first released from Matrigel by incubating in 2U/ml dispase (Life Technologies, Grand Island, NY) for 60 min at 37 °C and then trypsinized to obtain single cells. Four thousand cells were mixed in Matrigel and cultured as described above. Images were captured from the entire rim of each well and used to quantify the number, size, and percentage of labeled spheres in each culture.

### Flow Cytometry

For flow cytometry analysis, single-cell suspensions prepared from TdT-labeled SGs as described above were re-suspended in PBS-1% bovine serum albumin and stained with APC conjugated antibody against surface markers for 20 min at 4 °C in the dark. The antibodies include those against CD49f (clone GoH3, Cat# 50-112-3056 from eBioscience, San Diego, CA), c-Kit (clones 2B8 Cat# 17-1171-81 and ACK2 Cat# 17-1172-81 from eBioscience), CD24 and Sca1 (clones M1/69, Cat# 138505 and clone D7, Cat#108111 from BioLegend, San Diego, CA). Cells were washed with PBS-1% BSA and re-suspended in PBS-1%BSA containing 1:200 dilution of 7AAD viability dye (BioLegend) before being analyzed on FACSCaliber or FACSAria-III (BD Bioscience). Phycoerythrin (PE) channel was used to detect TdT.

### Immunofluorescent Staining

SMGs were harvested and the attached fat and connective tissues were dissected. One gland from each mouse was fixed in 4% paraformaldehyde at 4 °C for 1 hour, rinsed in PBS, and soaked in 30% sucrose before cryopreservation. Five micron cryosections were prepared, dried for at least 2 hrs, rehydrated, and immunostained as described previously^20^. The following primary antibodies were used: anti-GFP/YFP (chicken, 1:2000, Cat#ab13970 Abcam) for the R26R-YFP reporter strain, anti-c-Kit (clone 2B8,1:100, Cat# 14-1171-82, eBioscience), anti-Aqp5 (Rabbit, 1:100, Cat#178615, EMD Millipore, Billerica, MA), anti-K8 and K19 (Rat, 1:10, TROMA-I and –III, DHSB, University of Iowa), anti-K14 (Rabbit, 1:2000, Cat# PRB-155P, Covance, Berkeley, CA). Bound antibodies were detected with either Alexa-594 or Alexa-488 conjugated secondary antibodies (Molecular Probes, Eugene, OR), depending whether YFP or TdT were used for genetic labeling. TdT was detected by direct fluorescence. Sections were mounted in Vectashield® mounting medium containing 4′,6-diamidino-2-phenylindole (DAPI; Vector Laboratories, Burlingame, CA). Fluorescent staining was visualized using a Nikon E800 fluorescent microscope or a Leica TCS SP8X confocal laser scanning microscope. Image J was used for quantification of labeled cells using randomized images from a pool of at least 10 images taken at 400X from 3 different sections for each gland (from at least three mice).

### Statistical Analyses

All statistics including power analysis were performed using SPW12 software (Systat software Inc., San Jose, CA). When comparing two groups, the numbers of samples sufficient to result in statistically significant differences was determined using standard power calculations with α = 0.05 and a power of 0.8. Differences in means were evaluated by un-paired 2-sided Student’s t test when comparing two groups, and one way analysis of variance and the Tukey’s HSD post hoc comparison when comparing several groups. Significance was set at p < 0.05.

## Electronic supplementary material

Supplementary Data

## Data Availability

All materials, data and associated protocols promptly available to readers without undue qualifications in material transfer agreements.
