# Supplementary material for: c-Kit+ Cells in Adult Salivary Glands do not Function as Tissue Stem Cells
Source: Sci Rep. 2018 Sep 21;8:14193. doi: 10.1038/s41598-018-32557-1 (PMC6155036; doi:10.1038/s41598-018-32557-1)

**c-Kit^+^ Cells in Adult Salivary Glands do not Function as Tissue Stem Cells**

Mingyu Kwak^1^, Ninche Ninche^1^, Sabine Klein^2^, Dieter Saur^2^ and Soosan Ghazizadeh^1^*

**Fig. S1. Distribution pattern of TdT-labeled cells in the SMG.**

Representative images of submandibular glands of the pulse-labeled *c-KitCre^ERT2/+^*;RosaTdT mice immunostained for K8 and K19 (markers for ductal cells) and Aqp5 (a marker for acinar cells) showing restriction of TdT labeling to salivary ducts. Antibody staining is in green and TdT is in red. Both merged and single channels are shown. Blue nuclear staining is dapi. Scale Bars=50 μm. Arrow points to intercalated ducts. GD, granular ducts and Ac, acini.


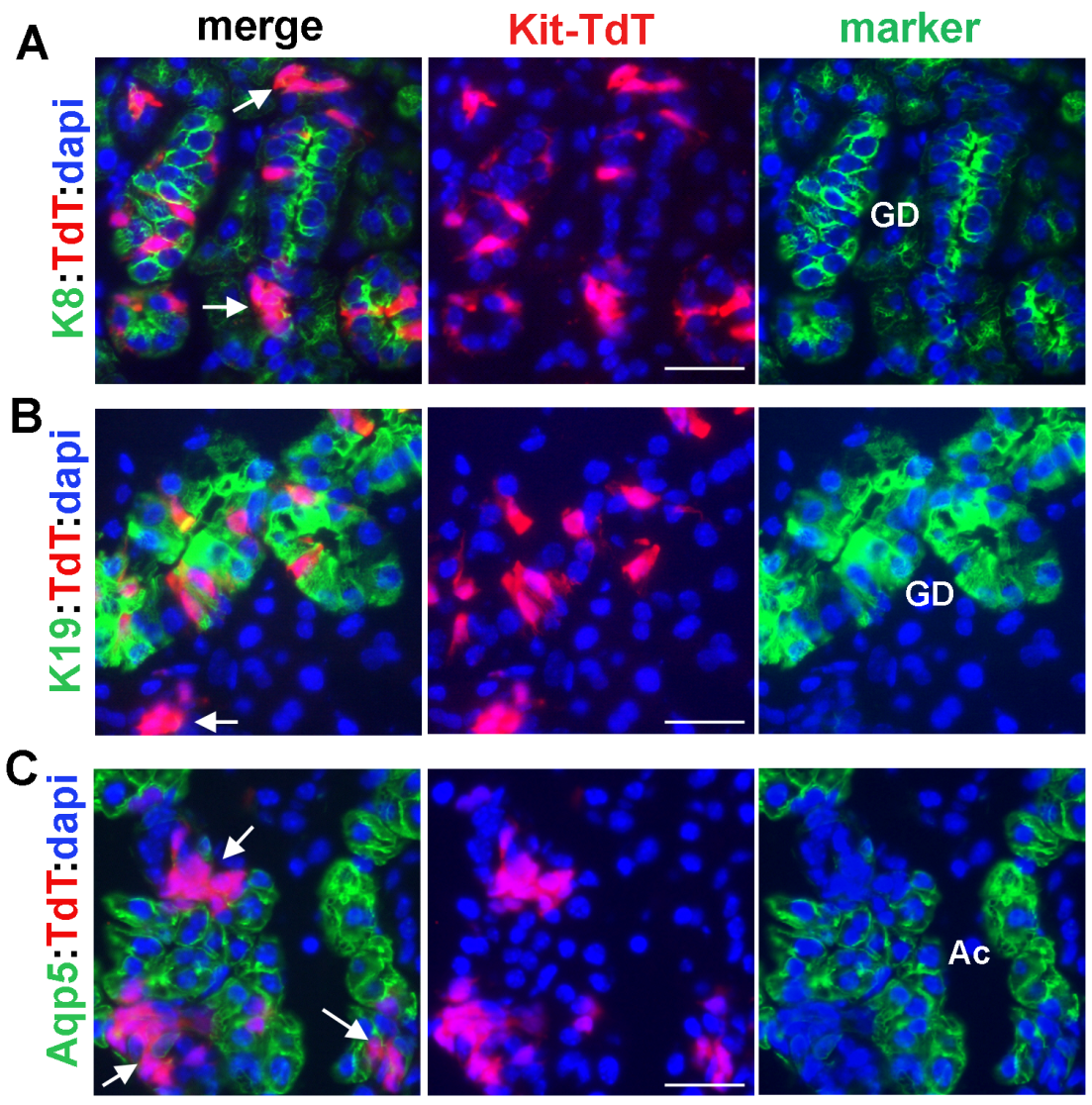


**Figure S2. Distribution of surface markers CD24 and Sca1 in the adult mouse SMG.**

Fluorescent images of mouse SMG stained for surface markers CD24 and Sca1. CD24 is expressed by all parenchymal cells while Sca1 is expressed in larger salivary ducts in addition to the vascular endothelium. Blue nuclear staining is dapi. Scale Bar=100 μm.


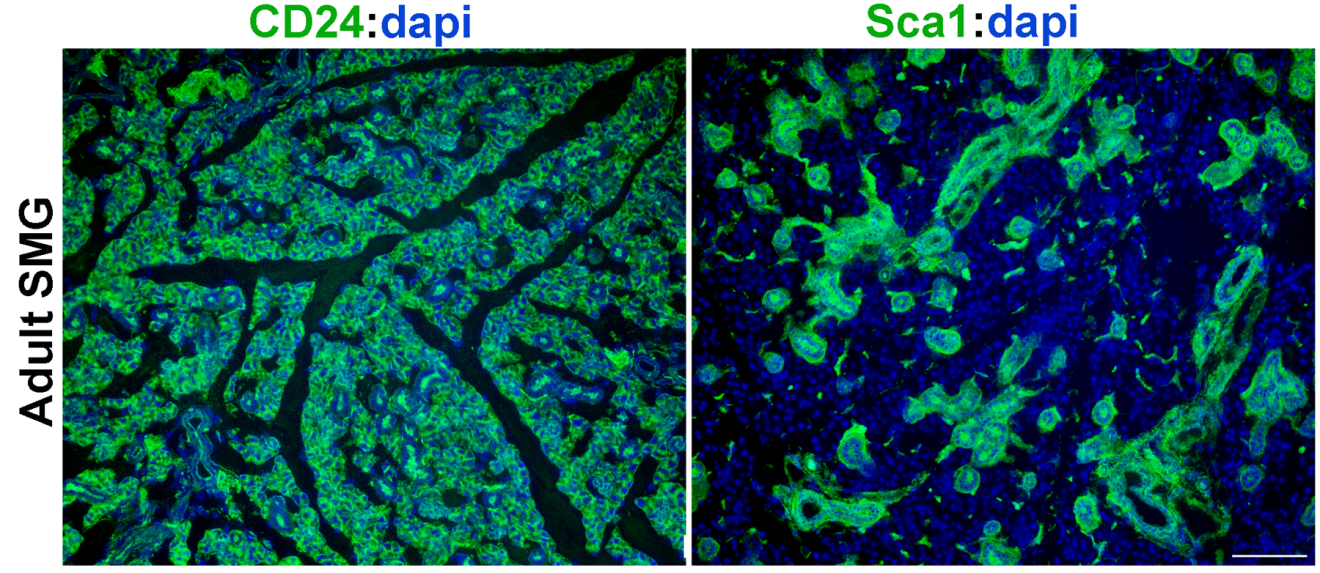


**Figure S3. Differential pattern of TdT labeling in major salivary glands of the same mouse.** Fluorescent images of parotid (Par), sublingual gland (SLG) and submandibular glands (SMG) harvested from c-KitCre^ERT2^:R26R-TdT mice 3 days after tamoxifen administration and stained for either Aqp5 or K14 (in green). Red fluorescence is TdT and blue nuclear staining is dapi. Scale Bar=50 um.


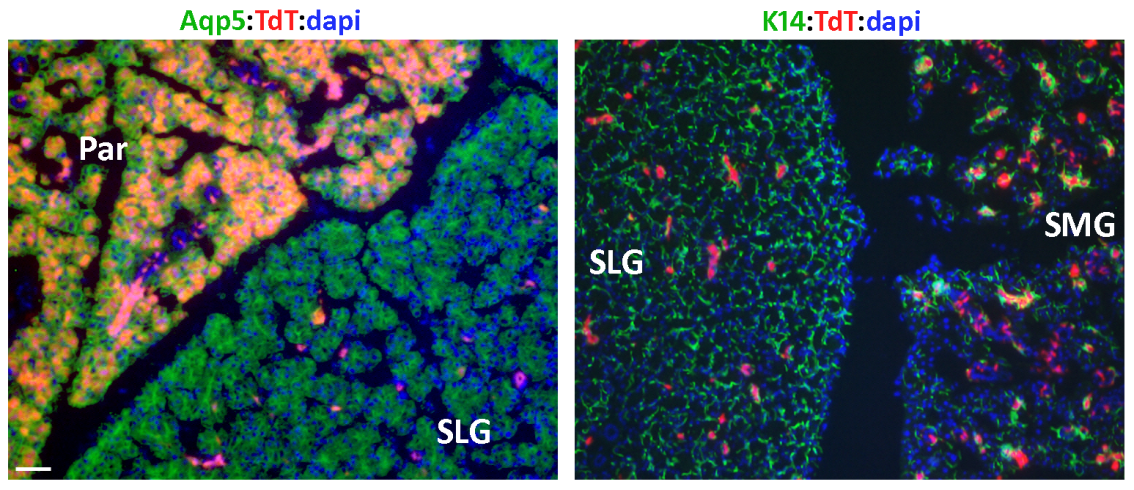


**Figure S4. Distribution and morphology of TdT-labeled c-Kit^+^ cells in the excretory and striated ducts.**

Confocal immunofluorescence images of cross sections of salivary ducts from TAM-treated c-KitCre^ERT2^:R26R-TdT mice. Tissues were stained either for K14, a marker for basal ED cells or for K19, a marker for differentiated ductal cells. Analysis of thin optical sections showed no overlap between K14^+^ and TdT^+^ cells. Arrows point to cells expressing both K19 and TdT. Arrowhead notes the basolateral projection of TdT^+^ tuft-like cells.


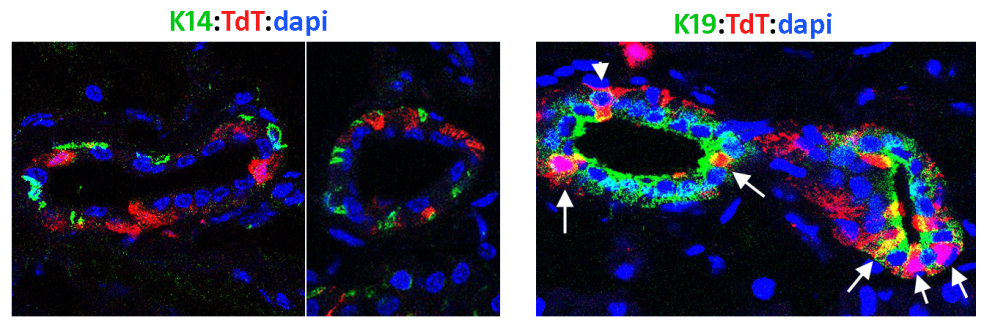


**Figure S5. Salisphere forming capacity of c-Kit^+^ cells.**

(A)Treatment of c-KitCre^ERT2^ mice with tamoxifen has no adverse effect on sphere forming capacity of SMG cells. Salispheres measuring >0.2 mm in diameter/10,000 SMG cells were quantified and values were expressed as mean+SEM. Differences are not significant.

(B) A representative phase/fluorescent image of secondary cultures established from SMG cells isolated from c-KitCre^ERT2^:R26R-TdT (n=5 independent cultures).


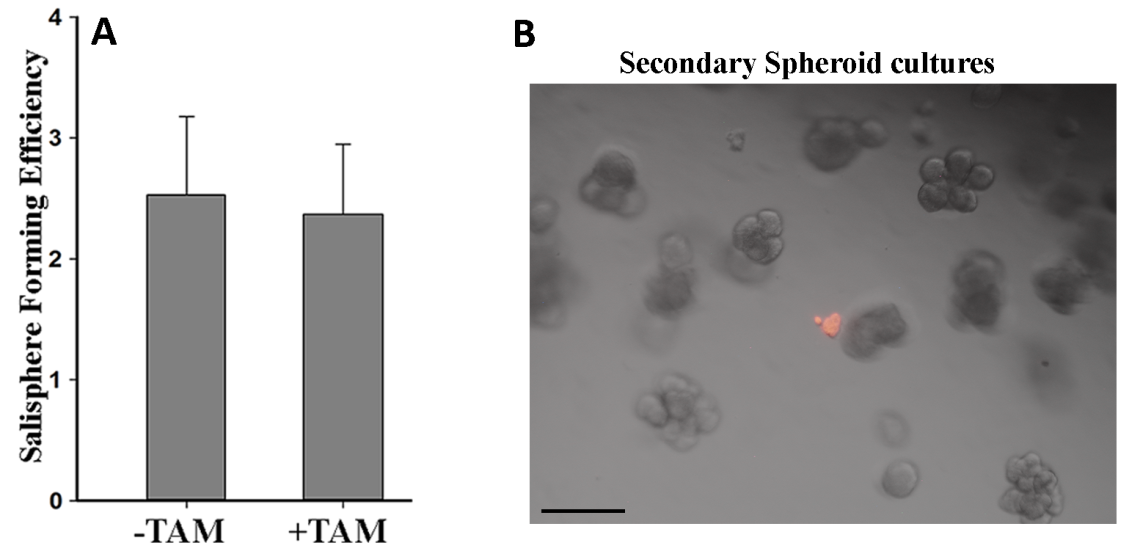


**Figure S6. Lineage tracing of c-Kit^+^ cells in adult SMG.** Representative images of SMG from c-KitCre^ERT2^:R26RYFP taken at low magnification to show the distribution of YFP-labeled cells over a 6 month period of chase. Sections were co-stained for YFP (green) and Aqp5 (red). Nuclear blue staining is dapi. Scale bar= 100 um.


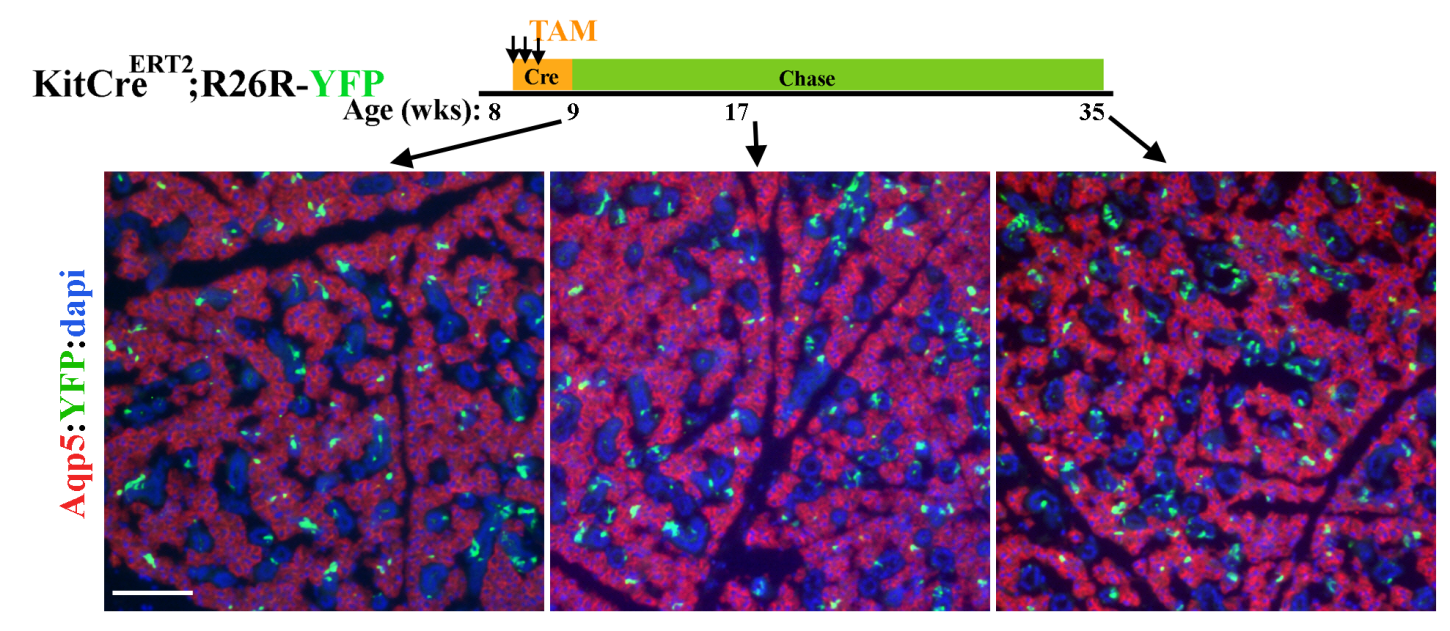

Supplement: Supplementary file 1 — Supplementary Data [file 41598_2018_32557_MOESM1_ESM.docx]
